# An outbreak of skin rash traced to a portable floating tank in Norway, May 2017

**DOI:** 10.2807/1560-7917.ES.2019.24.38.1900134

**Published:** 2019-09-19

**Authors:** Susanne Hyllestad, Heidi Lange, Bernardo Guzman-Herrador, Emily MacDonald, Vidar Lund, Preben Aavitsland, Line Vold

**Affiliations:** 1 Norwegian Institute of Public Health, Department of Zoonotic, Food- and Waterborne Infections,, Oslo, Norway; 2 University of Oslo, Faculty of Medicine, Institute of Health and Society, Oslo, Norway

**Keywords:** outbreak, floating tanks, skin rash, infection, folliculitis, waterborne infections, hygiene, infection control

## Abstract

Despite concerns about infection risks of floating tanks, outbreaks have rarely been reported. In May 2017, an outbreak of skin rash occurred among visitors of a floating tank open for the public in Norway. We assessed the extent and cause of the outbreak and the risk factors for infection in a retrospective cohort study among the visitors of the floating tank using a standardized web-based questionnaire. An environmental investigation was conducted including microbiological analysis of the floating tank water. Of the 46 respondents to the questionnaire (61 distributed), 22 reported symptoms, most commonly palmar and plantar rash, swollen lymph nodes, ear canal pain and itching. None of the investigated risk factors, such as sex, age, duration of bathing or use of the shower after bathing, were significantly associated with illness. The results of the environmental investigation indicated that the water was heavily contaminated by *P. aeruginosa* and heterotrophic bacteria. The outbreak investigation highlights the need to ensure adequate hygienic operation of floating tanks. Awareness about responsibilities should be raised among the operators of floating tanks and relevant operational parameters for floating tanks should be made available for local health authorities.

## Introduction

**Figure 1:**
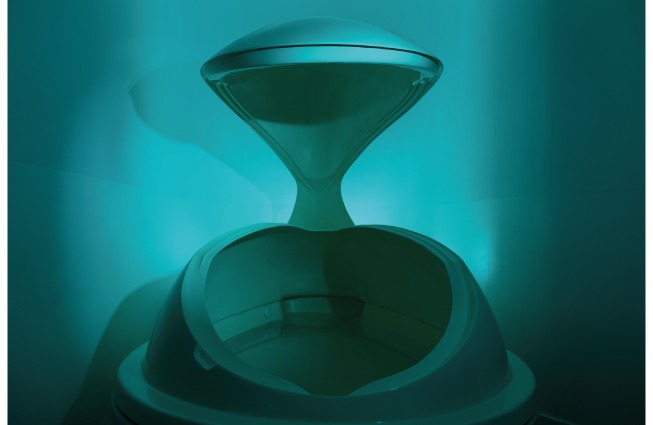
A typical floating tank for single-person use in commercial spas

A floating tank—also known as a float tank, floatation tank, isolation tank, immersion therapy tank, or sensory deprivation tank [1]—is a shallow pool filled with salt water, which allows the user to float with minimal effort, simulating a feeling of weightlessness [2]. A typical floating tank is 30–45 cm in depth and is filled with a near-saturated (25–30%) mixture of potable water and magnesium sulphate (MgSO_4_ or EPSOM salt) that is heated to 34–37 °C [2]. Floating tanks are often used in wellness or therapeutic contexts, mainly for single-person use (Figure 1) [2] and are an increasingly popular way to reduce stress through the Restricted Environmental Stimulation Technique (floatation-REST) [3].

Photo: Colourbox.com. Reproduced with permission from Kzenon.

Since first introduced in the United States (US) in the 1970s [4], commercial spas or salons with floating tanks have become widespread across Europe. Despite their growing popularity, floating tanks, as opposed to swimming pools and hot tubs, are largely unregulated in most countries [4]. Some examples of suggested operational routines exist, mainly in the US, Canada [1,4] and Germany [5]. According to commercial actors, manuals for management follow with the purchase of a floating tank. The high content of salt limits microbial growth in the floating tanks, but various disinfection practices are often used to maintain water quality, such as chlorine or ultraviolet lamps [1,2,4]. The water in the floating tank is usually continuously circulated via a treatment unit between users and should be regularly replaced with fresh water and newly added magnesium sulphate to reduce the risk of infection [1,2]. However, because replacing saturated salt water is expensive, it is typically only changed a few times per year [4].

In Norway, floating tanks are regulated by the Norwegian regulation for public pools and saunas. This requires that the floating tank is reported to the local health authorities before it opens to the public. However, specific guidelines for the management of floating tanks are not provided [6]. The regulation includes general requirements to ensure safe surroundings for all spa environments intended for public use, and specific requirements related to hygienic operation, including circulation of water, disinfection and water quality standards for swimming pools. Specific requirements are set for the water quality standards; free, available chlorine at a range of temperatures, physical (turbidity, pH) and microbiological quality (maximum 10 colony forming units (cfu)/mL of heterotrophic plate count at 37 °C and 0 cfu/100 mL *Pseudomonas aeruginosa*) [6]. None of the specific requirements refers to the quality of saline water.

Infection risks in floating tanks are a concern for national and local public health representatives because floating tanks have grown in popularity among the public and clear protocols for their inspection and operation are lacking [2,4]. The concern is mainly related to the presence of pathogenic bacteria owing to poor management of the floating tanks [2]. However, cases or outbreaks from visiting a floating tank have rarely been reported [2,4]. Against this backdrop, there have been calls for more knowledge on the management of floating tanks, both under normal and worst-case scenario operating conditions, to inform best practices [4].

In Norway, there have been reports of cases and outbreaks traced to whirlpools, but not to floating tanks [7]. Here, we describe an outbreak of skin rash traced to a portable floating tank in May 2017.

### Outbreak detection

In May 2017, in a municipality in south-east Norway, a member of the public informed the local health authority that that their family and another family had developed rashes after bathing in a floating tank at a temporary art exhibition at a gallery 3 or 4 days earlier. The same person reporting had visited the out-of-hours emergency room and received a preliminary diagnosis of *P. aeruginosa* infection. The local health authority notified the art gallery where the floating tank was on display as an art installation, and the gallery immediately closed access to the floating tank. The art gallery then contacted the ca 150 visitors to the floating tank whom they were able to identify. In collaboration with the local health authority, the art gallery informed the visitors about the infection risk and advised them to seek healthcare if they had developed symptoms. A similar message was also spread in the local news.

The local health authority was then contacted by several other art gallery visitors who had developed symptoms after visiting the floating tank. Based on the clinical picture, *P. aeruginosa* infections were suspected. On 7 June, the local health authority asked the Norwegian Institute of Public Health for support, and a joint investigation was initiated into the extent and cause of the outbreak and the risk factors for infection in order to implement control measures and inform future float tank operations. Here, we describe the results of this investigation.

## Methods

### Epidemiological investigation

We performed a retrospective cohort study among users of the floating tank for the period when the tank was available for visitors, which was between the opening of the exhibition (12 May 2017) and the day the tank was closed after detection of the outbreak (30 May 2017). As all the reported cases had been among the users of the tank, we did not include other gallery visitors.

The art gallery kept a list of visitors who had signed out towels and bathrobes before accessing the tank. Of these, 61 persons gave oral consent to be a part of the outbreak investigation when contacted by the art gallery administration. On 14 June 2017, the art gallery sent them the questionnaire link using SMS or email. No reminders were sent.

The web-based questionnaire was designed to collect information on demographics (age, sex, residency), use of the floating tank (date, duration, showering) and any symptoms (onset, duration, medical examination). The responder could tick off one or more of the following symptoms: rash/acne-like rash, swollen tender lymph nodes, rash under the feet or on the hands, itching, diarrhoea, stomach pain, fever, pain in the ear canals, ear infection or other symptoms (if an answer was yes, an open ended comment field was possible). The questionnaire ended with an open question about the respondent’s opinion about the cause of their symptoms.

The outcome of interest was disease. We defined a case of infection as a person who used the floating tank during the time period 12 to 30 May 2017 and who had within 14 days of visiting developed two or more of the following symptoms: rash/acne-like rash, swollen tender lymph nodes, rash under the feet or on the hands, itching, fever, pain in the ear canals or otitis externa. The symptoms were self-reported, but some reported that they had been diagnosed by a medical doctor.

Data from the survey were imported to Excel for data management and analysis. The data were analysed according to descriptive epidemiological factors such as demographics (mean and median of age, sex), illness characterisation and onset of the outbreak. We also compared the association between exposures (sex, age, length of immersion and showering) and disease. We calculated 95% exact confidence intervals using the mid-P method in Episheet.

### Environmental investigation

The local health authority inspected the floating tank on 30 May 2017, the same time when the tank was closed to the public. The personnel involved at the art gallery were interviewed regarding the history of the operation of the float tank, the established routines for its maintenance and their knowledge of existing relevant legislations. Because the exhibition changed the external firm advising them on swimming pool hygiene, water sampling started first a week after the closure of the floating tank. On 7 June, the art gallery personnel collected one 1,000 mL sample of water from each of the following sites: the floating tank, the adjacent shower built in the floating tank structure and from the showers in the changing rooms (located in the basement of the art gallery). The sampling equipment had been supplied by an accredited laboratory. Heterotrophic plate count and *P. aeruginosa* were chosen as agents for analysis because these have guideline values in the Norwegian regulation for public pools and saunas. The samples were analysed by an accredited laboratory for coliform bacteria and *E.coli* (according to ISO 9308–1:2014) by heterotrophic plate count (standard plate count) at 22 °C and 36 °C (according to ISO 6222:1999) and for *P. aeruginosa* in 100 mL (according to ISO 16266:2008).

### Ethical statement

Ethical approval was not needed because the Norwegian Institute of Public Health is able to access and use personal identifiable information for communicable disease outbreak investigations in the public interest. The questionnaires in this particular outbreak were distributed by the art gallery, and personal identifiers were not a part of the dataset. The persons invited to the outbreak investigation had given oral consent to the art gallery before the questionnaire was sent.

## Results

### Descriptive and analytical epidemiology of the outbreak

A total of 61 people who visited the floating tank were invited to participate in the outbreak investigation and 46 of them responded. Of the respondents, 29 were women, and the median age was 34 years (range: 6–80). Among the 46 respondents, 45 had used the floating tank during the study period. One of the responders reported not to have used the floating tank despite having signed out towel and bathrobe. We excluded this person from the analysis.

Of the 45 respondents who reported bathing in the floating tank, 22 fulfilled the definition for a case of infection. Eleven of them had sought healthcare. Four of those reported that their doctor in their respective local health area had taken microbiological samples from them; however, no results were made available to the Norwegian Institute of Public Health, during or after the outbreak investigation. As we did not have personal identifiers (only the gallery had), we could not pursue this further.

The most commonly reported symptoms among the 22 cases were a palmar or plantar rash (n = 14), swollen lymph nodes (n = 13), ear canal pain (n = 12) and itching (n = 11). The duration of symptoms was mainly between 7 and 14 days (Table 1).

**Table 1:** Characteristics and reported symptoms among cases, following bathing in a floating tank at an art exhibition, Norway, May 2017 (n = 22)

Characteristics of the suspected cases	Number of persons	%	95% CI
**Sex**			
Male	7	32	15–53
Female	15	68	47–85
**Age group (years)**			
0–19	7	32	15–53
20–59	14	64	42–81
≥ 60	1	5	0–20
**Duration of symptoms (days)**			
1–2	1	5	0–20
3–5	3	14	4–33
6–10	5	23	9–43
11–14	5	23	9–43
≥ 15	5	23	9–43
Symptoms present at the time of the survey	3	14	4–33
**Reported symptoms^a^**			
Rash/acne-like rash	14	64	42–81
Swollen, tender lymph glands	13	59	38–78
Pain in the ear canals	12	55	34–74
Itching	11	50	30–70
Other symptoms	7	32	15–53
Fever	6	27	12–48
Otitis	4	18	6–38
Rash under the feet or on the hands	3	14	4–33
Stomach pain	1	5	0–20
Diarrhoea	0	0	0–13

CI: confidence interval.

^a^ It was possible to report more than one option in the questionnaire.

**Figure 2:**
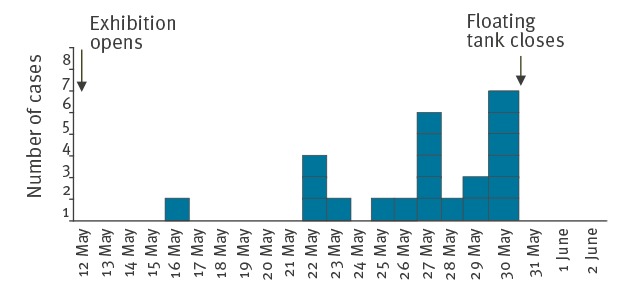
Epidemic curve of an outbreak of skin rash following bathing in a floating tank at an art exhibition, Norway, May 2017 (n = 21)

The median time from bathing to development of symptoms was 2 days (range: 0–4). Most cases occurred after the floating tank had been in operation for 9 days (Figure 2). The floating tank was closed on 30 May 2017.

One case was excluded from the epidemic curve because of inconsistent reporting of the time of bathing and time of developing symptoms.

None of the investigated risk factors were significantly associated with the infection (Table 2).

**Table 2:** Risk of infection following bathing in a floating tank at an art exhibition, Norway, May 2017 (n = 22)

Factors	Cases	Non-cases	Total	Attack rate in %	Risk ratio (95% CI)
All bathers	22	23	45	49	–
**Sex**					
Male	7	9	16	44	Ref
Female	15	14	29	52	1.2 (0.61–2.3)
**Age group (years)**					
0–19	7	6	13	54	1.1 (0.59–2.1)
20–59	14	15	29	48	Ref
≥ 60	1	2	3	33	0.7 (0.13–3.6)
**Duration of bathing (minutes)**					
1–10	15	19	34	44	Ref
≥ 11	7	4	11	64	1.4 (0.80–2.6)
**Showers used before and after bathing**					
In the inbuilt shower in the floating tank	2	0	2	100	2.4 (1.58–3.6)
In changing room	13	18	31	42	Ref
Both	7	5	12	58	1.3 (0.71–2.2)

CI: confidence interval; Ref: chosen as reference to estimate risk ratios within the category.

### Environmental investigation and inspection of the internal control systems

**Figure 3:**
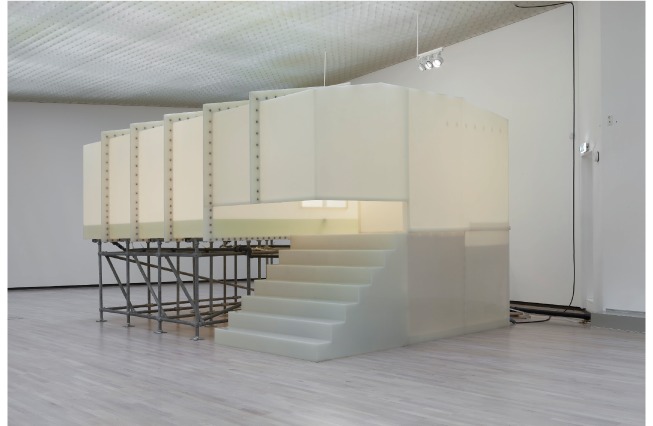
The floating tank from the exhibition, Norway, May 2017

The floating tank (Figure 3), created in 1999, was part of a temporary exhibition at the art gallery. The art gallery personnel and a consultant pool specialist installed the floating tank according to the supplied instruction manual. The floating tank was filled with fresh water from the municipal water supply system and salt (magnesium sulphate) was added before the opening of the exhibition. Personnel at the gallery were responsible for the management and routine maintenance of the floating tank. The pool specialist gave advice on salt and chlorination, including dose and monitoring. The exhibition period started on 12 May 2017 and lasted until 10 September 2017.

Photo reproduced with permission from Øystein Thorvaldsen/Henie Onstad Kunstsenter.

Visitors borrowed towels and bathrobes before entering the floating tank, and they could be immersed for up to 20 min with a maximum of four people at the same time. The art gallery personnel handled the registration of borrowed and returned towels and bathrobes. Towels and bathrobes were cleaned after each visitor, which was handled by a cleaning firm. The visitors were encouraged to shower before and after the float to avoid reaction with the salt, as described in the operation manual of the floating tank. Showers were available in the existing staff changing rooms in the gallery, in addition to a shower that was a part of the design of the floating tank. This inbuilt shower was connected to the municipal water supply (fresh water). According to the art gallery personnel, most users chose to use the showers in the changing rooms.

### Concentration of salt in the floating tank

At the opening of the floating tank to the public, the water in the tank had a salt concentration of ca 20%. A device to monitor the salt concentration was installed, but it was not in use during the operation of the floating tank. The personnel had assumed, based on advice from the external consultant, that a 20% salt concentration was sufficient to inhibit microbial growth. This assumption had not been checked by conducting microbiological analysis during the time the floating tank was in operation before the outbreak.

The personnel at the art gallery reported that, based on advice from the pool specialist, chlorine (5 g of granular calcium hypochlorite 70%) was added into the water system of the floating tank once a week, the amount logged in a manual and chemical analyses of the total and free, available chlorine were conducted once a week. However, on request, only two measurements of residual chlorine could be presented during the environmental investigation. On 26 May, the residual chlorine concentration was 0.02 mg/L and on 1 June, it was 0.01 mg/L. Both results are lower than the guideline value in the Norwegian regulation for public pools and saunas (0.9 mg/L at 34 °C for non-saline water) [6]. The measured levels of residual chlorine in the floating tank were not sufficient to deactivate bacteria in non-saline water. There is no guideline value for the concentration of free, available chlorine needed to inhibit growth in saline water.

### Microbiological analysis of water samples

The microbiological laboratory reported no growth of faecal bacteria (*E. coli*) from any water sample, but “massive growth” of both *P. aeruginosa* and heterotrophic bacteria at 22 and 36 °C in samples of the water from the floating tank and from the inbuilt shower in the floating tank. The designation “massive growth” implied that it was impossible to quantify the cfu, indicating heavy contamination. The results of the samples from the changing room showers in the basement were 9 cfu/mL heterotrophic bacteria at 22 °C, 15 cfu/mL heterotrophic bacteria at 36 °C and 3 cfu/100 mL *P. aeruginosa*, thus lower than in the floating tank areas.

### Outbreak control measures

The personnel at the art gallery lacked formal training in the operation of floating tanks and relied on advice from the external consultant. The floating tank was not reported to the local health authority since the personnel at the art gallery were not aware of the current legislation on public pools and saunas in Norway. When the floating tank was suspected as a source of the outbreak on 30 May 2017, it was immediately closed. Concurrently, the art gallery conducted a review of their operations of the floating tank and submitted necessary documentation to the local health authorities. The floating tank was re-opened on 4 July 2017. On 17 July, the art gallery contacted the local health authority about difficulties maintaining proper chlorine levels and reported that they had drained and cleaned the floating tank and refilled it with fresh water and added salt. They also reported that they restricted the number of visitors in the tank. The local health authorities emphasised measures for hygienic operation, including circulation of the water between users and daily exchange of the water with new salt added, to secure a stable level of residual chlorine. No further cases were reported after the re-opening of the floating tank.

## Discussion

We have described an outbreak of skin rash among the users of a floating tank in Norway in May 2017. Half of the visitors to the floating tank developed symptoms such as palmar or plantar rash, swollen lymph nodes, ear canal pain or itching. The environmental investigation revealed that the floating tank water and surroundings were heavily contaminated with *P. aeruginosa* and heterotrophic bacteria. The water in the floating tank had low levels of residual chlorine, lower than recommended salt concentration and the water was not replaced or circulated.

The findings of *P. aeruginosa* during the outbreak investigation added to the initial suspicion of the cause based on clinical observations made early in this outbreak. *P. aeruginosa* is an opportunistic pathogen reported in pool and spa environments [8-10]. Cases of folliculitis caused by *P. aeruginosa* were first described in the mid-1970s in association with exposure in a whirlpool [11-16] and later also in association with hot tubs [17], swimming pools [18], saunas [18] and water slides [19]. Typical manifestations of the infection include swimmer’s ear (otitis externa), hot tub rash (folliculitis) and hot foot syndrome (plantar skin eruptions) [8,19,20]. Both healthy and immunocompromised users may become infected, and symptoms may last up to 6 weeks [8]. The most frequently reported symptoms and duration of the infections in this outbreak were similar to other reported *P. aeruginosa* outbreaks [21-24], however, we have no conclusive evidence that *P. aeruginosa* was the cause of this outbreak. Both the lack of patient samples and a lack of microbiological analysis for other pathogens than *P. aeruginosa* represent limitations in the outbreak investigation. Similar symptoms of skin infection may be caused by other pathogens such as *Staphylococcus aureus*, *Mycobacterium* spp., *Streptococcus* spp., and *Acanthamoeba* spp. [9,10]. Although a relationship between pathogen and patients’ symptoms could not be established, we believe that the heavily microbiologically contaminated water in the floating tank caused the symptoms of the patients.

Inadequate operational routines as revealed by the environmental investigation may have led to conditions favourable to microbial growth [2,4], in particular a salt content at the lower end of the concentration (here approximately 20%) referred to as best practice [1], a lack of water circulation, and non-functional disinfection [2]. The recommended best practice for the salt content in floating tanks is 25–30%, which is near the saturation limit of magnesium sulphate (30% at 20 °C) [1]. This is a condition reported to inhibit growth of pathogenic microorganisms such as *P. aeruginosa*, *Candida albicans*, *Enterococcus faecium* and *Aspergillus niger* in water from floating tanks [25]. It is reported that there is only modest microbial activity at a salt concentration of approximately 20% [9]; however, microorganisms as *Pseudomonas*, *Vibrio* and *Micrococcus* spp. have been reported to tolerate maximum salt concentrations of 10–20% [26]. The concentration of salt in the floating tank was estimated at 20% when the tank was opened but because this assumption was not verified by monitoring, the actual salt concentration may have been lower. Minor amounts of water were refilled to keep the water level in the floating tank, and salt was added at the same time. However, the concentration of salt was not documented and it cannot be verified whether it was sufficient according to best practice.

Regular replacement of the water in floating tanks and circulation of the water after each visitor has been routinely highlighted as an effective measure to prevent microbiological growth in the floating tanks, along with disinfection measures [2,4]. The environmental investigation revealed that none of these measures had been in place for the floating tank in the outbreak. The chlorine content in the floating tank was too low to be effective (in the range of 0.01–0.02 mg/L). The personnel at the art gallery had not been trained to operate the floating tank and were not able to interpret the results of the testing conducted. However, they had been assured by the pool specialist that the results of the analysis were satisfactory. Despite operation according to guidelines and routines, pathogens such as *P. aeruginosa* have been detected in biofilms on the walls of pools [2]. This implies that circulation in combination with disinfection alone may not effectively prevent microbiological growth; physical cleaning is also required. Another preventive measure is showering before entering the floating tank in order to remove skin particles and other organic material that could prolong bacterial survival [2]. The high turnover of visitors (up to 12 persons/h were allowed) in the floating tank may also have contributed to the microbial growth, by increasing the load of organic material in the water. The personnel at the art gallery were convinced that their operation was hygienically safe, but they lacked the competence to establish proper routines.

There is a possibility that the *P. aeruginosa* detected in the floating tank have been transferred via a person visiting the floating tank [27]. There have been reports that – once introduced – pathogens may survive despite the saline environment [2]. If any *P. aeruginosa* (or other pathogenic microorganisms) had been introduced immediately after the opening of the floating tank on 12 May, it would probably have been inactivated by the saline environment in the floating tank. We believe that the deteriorated hygienic condition in the floating tank and the inbuilt shower caused the outbreak rather than a pathogen introduced via an infected person.

In the present outbreak, the risk of infection among those who bathed 11 min or longer was only slightly higher than those who bathed between 1 and 10 min, although the difference was not significant (relative risk (RR) = 1.4; 95% confidence interval (CI): 0.8–2.6). The dose–response relation of exposure to bathing water is not well understood; however, it has been hypothesised that the density of microorganisms implies a greater risk than the exposure time [28]. Those who showered in the changing room appeared to be more protected against infection than those who showered in the shower inbuilt in the floating tank where the water was heavily contaminated with microbial growth, (RR = 2.4; 95% CI: 1.58–3.6). However, since only two cases reported to have used only the inbuilt shower, and none of the non-cases, the data are too sparse to be conclusive.

There are several limitations in this outbreak investigation. Microbiological confirmation of the diagnosis in patients could have strengthened the investigation. Since we do not have results from patient samples, we do not know what caused the skin rash. As the environmental investigation only included *P. aeruginosa* and no other pathogens, the outbreak investigation was biased towards this pathogen and did not consider the possibility of other microorganisms that may cause skin infections. Another limitation is the lack of information about gallery visitors who had not been exposed to the floating tank. However, the suspicion that the floating tank was the source of the infection was strong and there is little reason to believe that there could have been other sources that could cause skin rashes (of similar magnitude) among the unexposed visitors. Owing to the small number of cases, the calculated attack rates may be by a result of chance and may not be valid as one value. 

The list of visitors in the floating tank was not complete (e.g. registering was not conducted every day, handwritten phone numbers were difficult to interpret). The lists kept for signing out towels and bathrobes were meant for other purposes that monitoring the visitors in the floating tank. This may have contributed to a suboptimal assessment of the total number of persons with symptoms corresponding to the case definition. Initially, the art gallery assumed that as many as 600–700 persons had visited the floating tank; however, they were only able to identify ca 150 visitors who had signed out towels and bathrobes. Of these 150 persons, ca 50 reported developing symptoms. Furthermore, the study population (n = 61) consisted of only those visitors who, when contacted by an art gallery representative, had given their oral consent to be invited to the outbreak investigation. We may have overestimated the attack rate since those who became sick may have had an interest in being a part of the investigation. 

In Norway, floating tanks are subject to the regulations concerning public pools and saunas; however, they do not contain relevant guidelines for floating tanks [6]. Since floating tanks are distinct from swimming pools, concerns have been raised related to operational factors such as disinfection and the circulation of the water [4]. Neither the art gallery personnel nor their hired external consultant were aware of the obligation to notify the municipality of the installation of the floating tank or of the Norwegian regulations for public pools and saunas. 

Although the management of this particular floating tank may not be representative of other floating tank owners, this outbreak situation serves as an example of how inadequate routine maintenance may result in a deterioration of hygienic condition in floating tanks. The floating tank had previously been installed in other galleries worldwide without similar situations being reported. However, infection related to swimming pools and spa environments is in general assumed to be under-reported [8].

## Conclusion

The nature and timing of the symptoms in users of the floating tank indicated early on in the outbreak that the infection was likely to be associated with exposure to contaminated water from the floating tank at the art gallery. The suspicion was further strengthened when the results from the microbiological sampling of the floating tank revealed that the water in the floating tank was heavily contaminated by *P. aeruginosa*. The closure of the floating tank was the most important outbreak control measure, and the cooperation and swift communication between the local health authorities and the art gallery limited the outbreak. Awareness of hygienic risks and existing regulations should be raised among establishments who offer floating tanks to the public. Guidelines for the safe and hygienic operation of floating tanks should be made available to local health inspectors.
